# Fear motivates and dread stalls: the role of emotions in climate support

**DOI:** 10.3389/fpsyg.2026.1667470

**Published:** 2026-02-12

**Authors:** Sarah Gradidge, Annelie J. Harvey, Nic Gibson, Helen Keyes, Alina Knuppel, Emily McKendrick, Rachel Ownsworth, Magdalena Zawisza

**Affiliations:** School of Psychology & Sport Science, Anglia Ruskin University, Cambridge, United Kingdom

**Keywords:** climate action, climate change, climate policy, emotion, state emotion

## Abstract

As the negative impacts of rapidly accelerating climate change increase in frequency and severity, widespread climate action in the population becomes increasingly urgent. The need for population-wide climate action and behavior change represents a significant psychological challenge that may be addressed through psychologically informed interventions. The current study investigates whether and how much 10 incidental state emotions (fear, dread, hope, anger, sadness, distress, worry, guilt, shame, and helplessness) contribute to climate change belief, climate policy support, and climate action in participants from the UK (*N* = 418). We report that greater fear and lower dread predict greater climate policy support, with no other state emotions predicting climate policy support. State emotions did not predict climate change belief or climate action. Our findings indicate that feeling fear, but not dread, may be important for climate policy support, yet such emotions may not translate into climate action. We discuss possible explanations for non-significant findings, such as a ceiling effect in climate change belief. Overall, our study uniquely explores the contributions of multiple incidental state emotions to climate action, indicating that nuanced differences in state emotions (e.g., fear vs. dread) may lead to different impacts on climate policy support.

## 1 Introduction

Consequences of human-caused climate change are being felt globally, with increasingly frequent and intense natural disasters, sea-level rise, food and water insecurity, species loss, and damage to ecosystems (Intergovernmental Panel on Climate Change [IPCC], 2023). Such phenomena will increasingly become the norm, as we are projected to exceed the Paris Agreement of a 1.5 °C rise in global warming by the 2030s (Intergovernmental Panel on Climate Change [IPCC], 2023). Six of nine ‘planetary boundaries’ are already breached (Richardson et al., 2023), which may ultimately create an environment hostile to human and non-human life (Richardson et al., 2023). Climate change is expected to cost the global economy $2,328 trillion between 2025 and 2,100, assuming no significant changes are implemented (‘business-as-usual’). It is still expected to cost $1,200 trillion even if 1.5 °C is not exceeded (Tiseo, 2024). Thus, the stakes are high, necessitating urgent, large-scale climate action.

Achieving climate action is largely a psychological issue, involving significant behavior change and a dramatic overhaul of high-carbon lifestyles (Stoddard et al., 2021). However, behavior change is notoriously difficult (Whitmarsh et al., 2021). This difficulty is due to logistical barriers, like ‘car dependence’ (Mattioli et al., 2020), and psychological barriers, such as uncertainty about how to change one’s lifestyle (Vieira et al., 2023). Extensive psychological interventions, drawing on cognitive (e.g., implementation intentions; Bell et al., 2016; Fennis et al., 2011; Holland et al., 2006; Rees et al., 2018), social (e.g., social norms; Cialdini and Jacobson, 2021; Schneider and van der Linden, 2023; Sparkman et al., 2020), and/or behavioral (e.g., nudging; Böhm et al., 2020; Song et al., 2023; Wee et al., 2021) processes, have been implemented. Research has explored whether climate action can be enhanced through ‘if, then’ implementation intentions, which theoretically help to automatize behavior, whereby ‘if’ people encounter certain situations (e.g., no longer using the TV), ‘then’ they engage in climate-friendly behaviors (e.g., fully turning off the TV; Bell et al., 2016). Additionally, as people are highly social, we may be influenced and/or informed by our peers to engage in climate action (social norms; Cialdini and Jacobson, 2021). Climate action may also be subtly encouraged through nudging by making climate-friendly behavior easier (e.g., making it the default) than climate-unfriendly behavior (Böhm et al., 2020).

However, despite extensive efforts, the effectiveness of psychological climate action interventions often remains low or is effective only for certain populations. For example, Vlasceanu et al. (2024), having tested 11 psychological interventions in a large cross-cultural sample (*N* = 59,440), found only small impacts of a few interventions (decreasing psychological distance, writing a letter to the future generation, negative emotion induction), which in turn did not inform actual behavior, whereas Berkebile-Weinberg et al. (2024) found a scientific consensus intervention to be effective only for political liberals. As we must urgently enhance climate action, efforts must therefore continue to develop more effective psychological interventions.

Less research has explored emotional (vs. cognitive, social, or behavioral) processes as drivers of climate action, yet emotions drive human behavior in multiple psychological phenomena (eating behavior, Devonport et al., 2019; consumer decisions, Sharma et al., 2023; risk-taking, Panno et al., 2013). Although behavior change models have traditionally neglected emotions (Shiota et al., 2023), emotions have more recently been incorporated into behavior change, with emotions being deliberately elicited and enhanced to encourage desired behaviors (‘emotion-leveraging’; Shiota et al., 2023). Research has often focused on emotion-related effects of climate change, such as eco-anxiety (Hickman et al., 2021; Lammel, 2025; Niedzwiedz and Katikireddi, 2023), but less on whether emotions drive climate change beliefs and action (Brosch, 2021; Brosch and Steg, 2021; Pihkala, 2022a). More recent research has explored emotions and emotion-related constructs such as climate distress as drivers of climate action (Brosch, 2025). For instance, higher climate change distress predicts greater climate activism (Latkin et al., 2022; Vercammen et al., 2023).

Research on specific emotions is mixed regarding whether each emotion informs climate action. For instance, fear is sometimes found to enhance pro-environmental behavior (von Gal et al., 2024; Yu and Lu, 2023), but not always (Ettinger et al., 2021), and, even when effective, fear appeals may not lead to sustainable behavior change (von Gal et al., 2024). Emotions with a similar valence to fear, like dread and worry, may predict environmental concern and support for environmental policies (Haltinner et al., 2021). However, research on this topic is limited. Anger seems to promote climate action (Stanley et al., 2021; von Gal et al., 2024), whereas research on sadness is mixed (Marczak et al., 2024; Pihkala, 2022b), perhaps due to varying ways of defining sadness (Pihkala, 2022b). Other research differentiates between “externalizing emotions” (anger, frustration, disgust, outrage, disappointment) and “approach emotions” (hope, interest, engagement, concern, and courage). Both “externalizing” and “approach” emotions may positively predict climate action. Conversely, “withdrawal emotions” (helplessness, disconnection, and isolation) may negatively predict climate action (Vercammen et al., 2023).

More internally focused negative emotions, like guilt, typically predict greater pro-environmental behavioral intentions (Harth et al., 2013; Hurst and Sintov, 2022; Shipley and van Riper, 2022). The impact of shame on climate action is relatively understudied. According to theory, shame may lead to psychological defensiveness, such as avoiding situations that remind oneself of environmental values (Aaltola, 2021). Yet, there is also a theoretical basis for shame being morally constructive, eliciting pro-environmental behavior (Aaltola, 2021).

Positive emotions, like hope, may be more effective in promoting behavior change (Cohen-Chen and Pliskin, 2025; Duncan et al., 2021; Geiger et al., 2023; Kim et al., 2021; Williamson and Thulin, 2022), as hope helps individuals anticipate positive future outcomes and remain committed to their goals (Cohen-Chen and Pliskin, 2025; Duncan et al., 2021). However, Ettinger et al. (2021) found no impact of hope on climate change perception or action.

Our understanding of the role of emotions in climate action is limited by several factors and limitations in research (e.g., Asutay et al., 2023; Kovács et al., 2024; Moreton et al., 2019). For instance, some research has measured trait rather than state emotions, whereby trait emotions are typically stable over time and across contexts within a particular individual, while state emotions are more context-dependent and changeable over time (Zelenski and Larsen, 2000). Trait emotions are less conducive to change and thus less suitable for interventions than state emotions (Kovács et al., 2024). There is also a tendency to measure climate action intentions rather than behavior, thus limiting ecological validity (Asutay et al., 2023; Kovács et al., 2024; Moreton et al., 2019). Another limitation is the lack of exploration of the relative contributions of each emotion to climate action. Previous research utilized correlation rather than regression (Moreton et al., 2019) or measured composite emotions (Asutay et al., 2023; Kovács et al., 2024). Most studies on emotion and climate action have also explored emotions either in isolation or in small groups (e.g., anxiety vs. fear, von Gal et al., 2024; anger and fear, Miller et al., 2009; guilt vs. pride, Hurst and Sintov, 2022; guilt vs. anger vs. pride, Harth et al., 2013; anxiety, anger, and sadness, Contreras et al., 2024; guilt vs. anger, Lu and Schuldt, 2015). Therefore, the development of effective climate action interventions is hampered by a lack of knowledge of which emotions to elicit.

As discussed above, study findings are mixed for some emotions, such as non-significant relationships between fear and climate action in some studies (Ettinger et al., 2021) and yet significant relationships between fear and climate action in others (von Gal et al., 2024; Yu and Lu, 2023). Thus, clarity is needed. Finally, studies, like those discussed earlier, have often measured climate-specific emotions (e.g., eco-anxiety), whereas less research has explored non-climate-specific emotions. Thus, emotions that occur with no connection to climate change are known as ‘incidental emotions’ (Lu and Schuldt, 2015). Thus, we have less understanding of whether general, ‘incidental’ emotions inform climate action than of climate-specific emotions.

The current study addresses the above gaps in the literature by measuring multiple incidental state emotions and their impact on climate action. This study is exploratory and therefore does not propose specific hypotheses, given the mixed literature on fear (Ettinger et al., 2021; von Gal et al., 2024) and the lack of prior research determining which emotions most strongly predict climate action. The study aims to (a) determine whether 10 incidental state emotions (fear, dread, hope, anger, sadness, distress, worry, guilt, shame, and helplessness) inform climate change belief and action (independent contributions), and (b) determine how much each of these incidental state emotions contributes to climate change belief and action (relative contributions).

## 2 Method

### 2.1 Participants

UK-based participants (*N* = 532) were recruited via Prolific. One hundred and fourteen participants were excluded for partial responses, leaving a final sample of 418 participants (50.7% male; *M*_age_ = 42.3, *SD*_age_ = 14.7, age range: 18–80), exceeding the sample size of 118 required per a power analysis (medium effect size, *α* = 0.05, power = 0.8, 10 predictors). Participants were reimbursed £3.

### 2.2 Materials

#### 2.2.1 Incidental state emotions

Participants were asked to what extent they currently feel each of the following 10 emotions on a Visual Analogue Scale from 0 ‘Not at all’ to 100 ‘Very much so’: ‘hopeful’, ‘fearful’, ‘angry’, ‘distressed’, ‘guilty’, ‘worried’, ‘dread’, ‘sad’, ‘shame,’ and ‘helpless’. Measurement of emotions as single items, rather than via scales, aligns with previous research (Bury et al., 2020; Moreton et al., 2019; Swim et al., 2022), and using Visual Analogue Scale measures of emotions allows more granularity and variation in data compared to Likert scales (Ahearn, 1997).

#### 2.2.2 Attention checks

To assess participants’ attention, two attention check items were included. These checks involved participants reading the text: ‘In the previous section, you viewed some information about climate change.’ To indicate you are reading this paragraph, please type the word 60 in the text box below, or select the color purple from a drop-down menu. No participants failed either attention check.

#### 2.2.3 Climate change belief

Climate change belief was measured using four items (Vlasceanu et al., 2024), with participants indicating how accurate they thought four statements were on a scale from 0 ‘not at all accurate’ to 100 ‘extremely accurate.’ The statements were ‘Taking action to fight climate change is necessary to avoid a global catastrophe,’ ‘Human activities are causing climate change,’ ‘Climate change poses a serious threat to humanity,’ and ‘Climate change is a global emergency.’ No items were reverse-scored, with items summed to create a total climate change belief score. Higher scores indicate greater belief in climate change, with a maximum score of 400. The scale had adequate reliability within the current study, *α* = 0.95.

#### 2.2.4 Climate policy support

Climate policy support was measured using nine items, each describing a climate policy (Vlasceanu et al., 2024), with participants indicating their agreement with each policy from 0 ‘not at all’ to 50 ‘moderately’ to 100 ‘very much so.’ The policy items were ‘Raising carbon taxes on gas/fossil fuels/coal,’ ‘Significantly expanding infrastructure for public transportation,’ ‘Increasing the number of charging stations for electric vehicles,’ ‘Increasing the use of sustainable energy such as wind and solar,’ ‘Increasing taxes on airline companies to offset carbon emissions,’ ‘Protecting forested and land areas,’ ‘Investing more in green jobs and businesses,’ ‘Introducing laws to keep waterways and oceans clean,’ and ‘Increasing taxes on carbon-intensive foods (for example, meat and dairy).’ Participants could answer ‘not applicable’ to any policy, leaving a blank score for that policy item. No items were reverse-scored, with items summed to create a total climate policy support score. Higher scores indicate greater climate policy support. The scale had adequate reliability within the current study, *α* = 0.87.

#### 2.2.5 Climate action

Climate action was measured using the Work for Environmental Protection Task (WEPT; Lange and Dewitte, 2022), used as a proxy for climate action. Participants were informed that engaging in the task would result in one tree being planted for every page of the task they complete, up to a maximum of eight pages, with 60 numbers per page. Participants completed a two-digit number identification exercise in which they ticked all numbers with an even first digit and odd second digit (e.g., 27). Participants could opt to end the task at any page. The task is intentionally designed to be tedious, to measure, in behavioral terms, how willing participants are to complete this task (i.e., endure tedium) to help the environment. The task is not identical to real-world climate action. However, the task does share similarities with real-world climate action: It measures effortful behavior that is not personally rewarding (and may incur personal disadvantages, e.g., tedium) but that has pro-environmental impacts. This task serves as a proxy for climate action in an online survey (Lange and Dewitte, 2022). Each completed page is coded as 1, and non-completed pages are coded as 0, with the number of completed pages summed for a total WEPT score. A higher WEPT score is considered a stronger behavioral indicator of willingness to help the environment. The external validity of the task is supported by evidence that WEPT performance is influenced by the magnitude of costs (e.g., the number of items to complete) and benefits (e.g., the amount of pro-environmental donation given), suggesting participants perceive costs and benefits as real (Lange and Dewitte, 2022).

### 2.3 Design

The study follows a multiple linear regression design, with the 10 incidental state emotions as predictors, and climate change belief, climate policy support, and climate action as outcome variables in three separate regressions. Gender was included as a covariate.

### 2.4 Ethics statement

The study received ethical approval from the Anglia Ruskin University School of Psychology and Sport Science Research Ethics Panel (code: ETH2122-2386).

### 2.5 Procedure

Participants registered for the study via Prolific before being redirected to the survey on Qualtrics. Participants read the participant information sheet and provided informed consent. As this study formed part of a larger cross-cultural Many Labs study that explored the effectiveness of 11 psychological interventions (plus control) on climate action (Vlasceanu et al., 2024), participants were exposed to one of 11 interventions or control. These interventions are not discussed further in this study and were not included in the analyses, as they were not the core focus of our study. Small sample sizes per intervention in our sample (*N*s = 28–38) would have resulted in underpowered analyses. Further information on the interventions can be found in Vlasceanu et al. (2024). After reading their intervention, participants completed the first attention check before answering the outcome variable measures (climate change belief, climate policy support, WEPT) presented in randomized order.

Participants then provided demographic information (gender, age, education level, political orientation, and socioeconomic status) and answered the second attention check. The incidental state emotion measures were then completed at the end of the survey. Finally, participants were debriefed and redirected back to Prolific to receive reimbursement.

## 3 Results

All analyses were conducted using Jamovi. All statistical assumptions were met or resolved. Collinearity between the emotions was acceptable for all predictors (VIFs = 1.14–3.88). As gender has previously been linked to climate change belief and action (Elert and Lundin, 2022), and demographic variables should be included as covariates when theoretically or statistically justified (Tabachnick and Fidell, 2019), we included gender as a covariate. Gender was dummy-coded with women as the reference group (coded as zero). We did not include any other demographics (e.g., age) as covariates, as research is less consistent on the relationships between these demographics and climate change belief and action.

### 3.1 Climate change belief

Incidental state emotions significantly predicted climate change belief, *F*(11, 406) = 3.3, *p* < 0.001, accounting for 5.7% of variance in belief (adj. *R*^2^ = 0.057). However, no individual incidental state emotion significantly predicted climate change belief, *p*s > 0.05. Climate change belief was high among participants (*M* = 339, *SD* = 80.1), so we discuss a possible ceiling effect for this measure. Gender significantly predicted climate change belief (*β* = −0.259), *p* = 0.007, whereby women had greater climate change belief than men. Table 1 provides the full results of this regression.

**Table 1:** Multiple regression of 10 incidental state emotions on climate change belief.

Predictor	*β*	*B*	SE	*t*	*p*
Hopeful	−0.048	−0.145	0.153	−0.948	0.344
Fearful	0.173	0.507	0.274	1.848	0.065
Angry	0.001	0.005	0.245	0.019	0.985
Distressed	−0.014	−0.049	0.298	−0.165	0.869
Guilty	0.068	0.259	0.302	0.858	0.392
Worried	0.084	0.227	0.248	0.916	0.36
Dread	−0.142	−0.437	0.287	−1.52	0.129
Sad	0.057	0.167	0.25	0.67	0.503
Shame	−0.029	−0.114	0.332	−0.345	0.73
Helpless	0.043	0.121	0.243	0.5	0.618
Gender	−0.259	−20.724	7.695	−2.693	0.007

### 3.2 Climate policy support

Incidental state emotions significantly predicted climate policy support, *F*(11, 406) = 3.09, *p* < 0.001, accounting for 5.2% of variance in climate policy support (adj. *R*^2^ = 0.052). Feeling fearful (*β* = 0.202) and feeling dread (*β* = −0.244) significantly predicted climate policy support, *p*s < 0.05, with those who were more fearful reporting higher climate policy support, and those with greater dread reporting lower support. Gender significantly predicted climate policy support (*β* = −0.19), *p* = 0.049, such that women had greater climate policy support than men. Table 2 provides the full results of this regression.

**Table 2:** Multiple regression of 10 incidental state emotions on climate policy support.

Predictor	*β*	*B*	SE	*t*	*p*
Hopeful	0.038	0.217	0.291	0.747	0.455
Fearful	0.202	1.122	0.521	2.155	0.032
Angry	0.086	0.541	0.466	1.16	0.247
Distressed	−0.027	−0.176	0.565	−0.311	0.756
Guilty	0.023	0.165	0.574	0.287	0.775
Worried	0.026	0.13	0.47	0.277	0.782
Dread	−0.244	−1.419	0.546	−2.599	0.01
Sad	0.08	0.448	0.474	0.944	0.346
Shame	−0.003	−0.023	0.63	−0.036	0.971
Helpless	0.107	0.576	0.462	1.248	0.213
Gender	−0.19	−28.872	14.617	−1.975	0.049

### 3.3 Climate action

Incidental state emotions did not significantly predict climate action, *F*(11, 406) = 1.44, *p* = 0.154, adj. *R*^2^ = 0.011. We report the full results of this regression in Table 3.

**Table 3:** Multiple regression of 10 incidental state emotions on climate action.

Predictor	*β*	*B*	SE	*t*	*p*
Hopeful	0.076	0.009	0.006	1.463	0.144
Fearful	−0.1	−0.012	0.012	−1.041	0.298
Angry	0.165	0.023	0.01	2.183	0.03
Distressed	0.047	0.007	0.013	0.527	0.598
Guilty	−0.032	−0.005	0.013	−0.39	0.697
Worried	0.142	0.016	0.01	1.502	0.134
Dread	−0.155	−0.02	0.012	−1.614	0.107
Sad	0.071	0.009	0.011	0.816	0.415
Shame	−0.088	−0.014	0.014	−1.023	0.307
Helpless	−0.54	0.006	0.01	0.609	0.543
Gender	0.046	0.152	0.325	0.466	0.641

## 4 Discussion

The current study uniquely explored the independent predictive power of 10 incidental state emotions for climate change belief, climate policy support, and climate action. In this study, no incidental state emotions predicted climate change belief, but most participants agreed that climate change is occurring, indicating a ceiling effect for climate change belief. No incidental state emotions predicted WEPT performance, as a proxy of climate action. However, two incidental state emotions (fear and dread) significantly predicted climate policy support, with greater fear predicting higher support and greater dread predicting lower support. Therefore, feeling fearful, but not dread, may inform climate policy support. One interpretation of this finding conceptualizes dread as akin to intense fear. Early research indicated an inverted-U-shaped relationship between fear intensity and behavior change, whereby very weak or very strong fear appeals failed to enact behavior change, presumably due to insufficient motivation or a defense mechanism, respectively (Leventhal et al., 1967). The Extended Parallel Process Model (Maloney et al., 2011; Witte, 1992) integrated decades of fear appeal research, finding that response to fear may depend on the interaction between perceived threat and perceived self-efficacy in averting the threat, causing either protective motivation and message acceptance (danger control process) or defensive motivation and message rejection (fear control process). We postulate that fear may enhance motivation, leading to greater climate policy support, while dread (if interpreted as intense fear) may decrease motivation, causing lower climate policy support. This finding adds to previous mixed findings on fear and pro-environmental behavior, whereby fear enhances climate action in some studies (von Gal et al., 2024; Yu and Lu, 2023), but not others (Ettinger et al., 2021). Thus, the current study suggests fear intensity may inform climate policy support, aligning with some research (e.g., Tannenbaum et al., 2015). Our findings suggest that fear should be carefully measured to ensure it is separated from dread and related emotions, and that fear intensity should be measured and controlled when considering the role of fear in climate policy support.

### 4.1 Practical applications and policy implications

Our findings indicate that among the 10 incidental state emotions measured, increased incidental state fear and decreased incidental state dread may most effectively motivate support for climate policy. Therefore, interventions aimed at promoting climate policy support may need to enhance fear while avoiding dread. Interventions would require pretesting to ensure optimal fear. Further, theoretical models should inform the necessary steps to increase the effectiveness of fear appeals. For example, the ordered protection motivation model (Tanner Jr et al., 1991) suggests successful fear appeals must include three sequential steps: trigger fear, show the causes of this fear, and provide easy solutions to eliminate the threat. Pretesting should also assess fear intensity in the target audience to account for individual differences such as fear sensitivity, gender, age, and social identity (Zheng, 2020). As neither fear nor dread significantly predicted WEPT performance, as a proxy of climate action, translating fear into action may require addressing multiple barriers to behavior change, as outlined by the COM-B framework (Michie et al., 2011). These barriers include capability (physical and psychological ability to enact climate actions), opportunity (external factors that enable climate action), and motivation (internal processes that drive climate action above and beyond fear). Ultimately, a multiprong approach is needed to enhance climate action, with fear being a useful motivator.

### 4.2 Limitations and suggestions for future research

To measure climate change beliefs, participants rated the perceived accuracy of four statements about climate change, including those highlighting the threat and human contribution to climate change. There has been a scientific consensus for decades that human-generated carbon emissions are affecting the climate (Cook et al., 2016), and the issue has been widely publicized. It is perhaps unsurprising then that we found high agreement on the statements measuring climate change belief, with a mean of 339 (*SD* = 80.1) out of a maximum possible score of 400. Our data for this variable were negatively skewed (−2.13) and leptokurtic (5.04), and 25.4% of our sample scored the highest score of 400, indicating a ceiling effect (whereby a ceiling effect is defined as 15% of the data at the highest score, Lim et al., 2015; Wang et al., 2008). This ceiling effect reduces interpretive power as items in this measure may primarily capture perceived threat and urgency rather than causal belief. Future research may employ more granular measures of climate change belief, allowing for a greater spread of scores and a clearer assessment of any relationship between emotions and climate change belief.

We used the WEPT as a proxy of climate action, whereby this task indicates climate action by asking participants to complete a laborious number-identifying task, with more pages completed equaling more trees being planted. While studies have supported the WEPT’s validity (Lange and Dewitte, 2022, 2023), others suggest it weakly correlates with other behavioral tasks and does not fully align with pro-environmental behavior (Bosshard et al., 2024). Although the WEPT indicates willingness to engage in climate action through time-consuming behavioral effort, it does not capture other means people might use to act pro-environmentally, such as using finances (e.g., Berger and Wyss, 2021). Therefore, emotions may not predict climate action when the required resource is time, but research has yet to consider whether emotions predict climate action that utilizes financial resources. We encourage future research to utilize various behavioral tasks to capture climate action.

Another limitation is that our study explored discrete emotions (i.e., emotions in isolation), without considering the overlap and coexistence of emotions, including contradictory emotions (e.g., fear and hope). Although state emotions are typically more discrete than trait emotions (Zelenski and Larsen, 2000), coexisting incidental state emotions may moderate each other’s impact on climate belief and action. Thus, our findings, whereby greater fear predicts greater climate policy support and greater dread predicts less policy support, may depend on concurrent emotions. Future research should therefore measure coexisting incidental state emotions, not just discrete emotions.

Further, our sample was UK-based and middle-aged, limiting the generalizability of our findings to other age groups and nations. For example, young people can experience greater eco-anxiety than older people (Lammel, 2025) as they are more likely to be negatively affected by climate change in their lifetime. Countries also differ in eco-anxiety, with the Philippines and India particularly high, and the UK and USA lower (Hickman et al., 2021). Hence, fear appeals may work better as a motivator in the latter countries and older samples, and less so in younger and more eco-anxious nations, where fear appeals may instead trigger dread.

Finally, our study is correlational, and we recommend that future studies employ an experimental design to capture any causal impact of fear and dread on climate policy support.

### 4.3 Conclusion

The current study explored 10 incidental state emotions to test their independent and relative contributions to climate change belief, climate policy support, and climate action. Findings indicated that feeling incidental state fear, but not incidental state dread, is key for enhancing climate policy support, though future research must test causality. Incidental state emotions did not predict climate change belief or climate action, perhaps due to a ceiling effect on climate change belief and difficulty in bringing about behavior change. Our study suggests interventions designed to garner climate policy support should utilize fear, while avoiding dread. The ceiling effect for climate change belief in our UK sample indicates a majority belief among lay people, suggesting real-world interventions are not typically needed to enhance climate change belief. Effective psychological interventions to encourage climate action remain elusive. We encourage future research that explores real-world climate action beyond the WEPT.

## Data Availability

The dataset presented in this study can be found in the OSF online repository. The name of the repository and accession number can be found here: https://osf.io/swgxp?view_only=32732aa1095d4edebca88515b16b125e.
